# A Multicenter Real‐World Retrospective Study for Brentuximab Vedotin, Cyclophosphamide, Doxorubicin, and Prednisolone for Previously Untreated Patients With CD30‐Positive Adult T‐Cell Leukemia‐Lymphoma

**DOI:** 10.1002/hon.70141

**Published:** 2025-10-21

**Authors:** Masahito Tokunaga, Junya Makiyama, Motoaki Shiratsuchi, Takanori Toyama, Satoshi Oka, Ilseung Choi, Takahiro Yoshida, Kiyoshi Okazuka, Atae Utsunomiya

**Affiliations:** ^1^ Department of Hematology Imamura General Hospital Kagoshima Japan; ^2^ Department of Hematology Sasebo City General Hospital Sasebo Japan; ^3^ Department of Hematology Iizuka Hospital Iizuka Japan; ^4^ Department of Internal Medicine Miyazaki Prefectural Nobeoka Hospital Nobeoka Japan; ^5^ Department of Hematology and Blood Transfusion Kochi Health Sciences Center Kochi Japan; ^6^ Department of Hematology and Cell Therapy NHO Kyushu Cancer Center Fukuoka Japan; ^7^ Japan Medical Affairs Japan Oncology Business Unit Takeda Pharmaceutical Company Limited Tokyo Japan

**Keywords:** adult T‐cell leukemia‐lymphoma, brentuximab vedotin, PTCL, retrospective studies

## Abstract

Adult T‐cell leukemia‐lymphoma (ATL) is a rare, aggressive malignancy prevalent in Japan, the Caribbean, and Central/South America. This multicenter retrospective study evaluated the effectiveness and safety of brentuximab vedotin with cyclophosphamide, doxorubicin, and prednisolone (BV‐CHP) in patients aged ≥ 18 years with previously untreated CD30‐positive ATL, verified through immunohistochemistry/flow cytometry, from six hospitals in Japan. Outcomes included overall response rate (ORR; primary outcome), overall survival (OS), progression‐free survival (PFS), complete response rate (CRR), disease control rate (DCR), and safety. Subgroup analyses evaluated lesion site, ATL subtype, age, and CD30 expression. Of 46 screened patients, 36 (median age 71 years; 66.7% female) were analyzed and started BV‐CHP between April 2020 and January 2024. CD30 positivity was confirmed in all patients. ORR was 86.1% (95% confidence interval [CI] 70.5–95.3), CRR 61.1% (95% CI 43.5–76.9), and DCR 91.7% (95% CI 77.5–98.3). ORR by lesion site (lymph nodes, peripheral blood, skin) was 93.8%, 90.9%, and 83.3%, respectively, by ATL subtype (acute, lymphoma) was 78.9% and 94.1%, respectively, and by age (≤ 70 years, > 70 years) was 84.6% and 87.0%, respectively. One patient with CD30 expression < 10% achieved a complete response; ORR was 73.7% in 19 patients with CD30 expression ≥ 10%. Median OS and PFS was 535 days (95% CI 343–not estimable) and 205 days (95% CI 166–279), respectively. Treatment‐emergent adverse events of any grade and grade ≥ 3 both occurred in 88.9% of patients, with neutropenia, febrile neutropenia, and thrombocytopenia being most common. Among 11 patients who underwent allogeneic stem cell transplantation, two developed acute graft‐versus‐host disease; median PFS was 234 days (95% CI 168–343), compared with 180 days (95% CI 96–279) without transplantation. BV‐CHP demonstrated high ORR and CRR across age groups and ATL subtypes with a manageable safety profile, supporting its potential use as a standard treatment option.

## 1 Introduction

Adult T‐cell leukemia‐lymphoma (ATL) is a peripheral T‐cell lymphoma (PTCL), caused by human T‐cell leukemia virus type I (HTLV‐1) [1, 2, 3, 4]. HTLV‐1 is endemic in several regions, including Japan, the Caribbean, Central and South America, intertropical Africa, and the Middle East [5, 6, 7]. Globally, it is estimated that 5–20 million people are infected with HTLV‐1 [7, 8], with approximately 658,000 carriers in Japan alone [9]. It is well known that 3%–5% of HTLV‐1 carriers eventually develop ATL following decades of viral latency [10, 11].

Clinically, ATL is classified into four subtypes: smoldering, chronic, lymphoma, and acute [12]. Of these, the unfavorable chronic, lymphoma, and acute subtypes are considered aggressive forms, with a median overall survival (OS) for lymphoma and acute subtypes ranging from 6.2 to 10.6 months in real‐world studies [12, 13, 14]. Patients with unfavorable chronic‐type ATL are defined as those with at least one of the following three factors: serum lactate dehydrogenase or blood urea nitrogen levels greater than the upper limit of normal, or serum albumin levels less than the lower limit of normal [15].

As per the National Comprehensive Cancer Network guidelines, standard treatment regimens for ATL include brentuximab vedotin combined with cyclophosphamide, doxorubicin, and prednisone (BV‐CHP) for CD30‐positive cases, dose‐adjusted EPOCH (etoposide, prednisone, vincristine, cyclophosphamide, and doxorubicin), and zidovudine with interferon for acute, chronic, and smoldering subtypes [16, 17]. In Japan, the JCOG9801 trial compared the modified LSG15 (mLSG15) regimen—consisting of (1) vincristine, cyclophosphamide, doxorubicin, and prednisone; (2) doxorubicin, ranimustine, and prednisone; and (3) vindesine, etoposide, carboplatin, and prednisone—with biweekly cyclophosphamide, doxorubicin, vincristine, and prednisone (CHOP) in previously untreated patients with aggressive ATL [18]. Based on the promising results of JCOG9801, mLSG15 is considered [19] as a standard regimen. Mogamulizumab is a humanized anti‐CC chemokine receptor‐4 (CCR‐4) antibody, and CCR‐4 is expressed in approximately 90% of patients with ATL [20]. Mogamulizumab has been combined with mLSG15 [21] and CHOP [22]. Although the combination strategies with mogamulizumab showed higher efficacy than chemotherapy without mogamulizumab, several grade ≥ 3 adverse events, such as skin disorders, cytomegalovirus infection, and pyrexia, were observed only in the mogamulizumab plus mLSG15 arm [21]. Meanwhile, the ECHELON‐2 trial demonstrated that BV‐CHP significantly improved progression‐free survival (PFS), OS, and overall response rate (ORR) in patients with previously untreated CD30‐positive PTCL when compared with CHOP, but only four patients with ATL were included in the BV‐CHP group, limiting the applicability of these findings to ATL [23].

Given the scarcity of data on BV‐CHP for ATL, we conducted a multicenter, real‐world retrospective study to evaluate the effectiveness and safety of BV‐CHP in patients with previously untreated CD30‐positive ATL.

## 2 Materials and Methods

### 2.1 Study Design

This retrospective, multicenter observational study was conducted to evaluate the effectiveness and safety of BV‐CHP in patients with untreated CD30‐positive ATL in Japan. Clinical data were retrospectively collected from the medical records of patients across six participating hospitals where BV‐CHP was prescribed for newly diagnosed ATL. CD30 positivity was confirmed at each institution through immunohistochemistry (IHC) or flow cytometry (FCM). Overall best response were collected from BV‐CHP initiation to 4 weeks after discontinuation/termination. Adverse events (AEs) were collected during the same period or before post‐treatment initiation, whichever occurred first.

The study was conducted in accordance with the Ethical Guidelines for Medical and Biological Research Involving Human Subjects. Informed consent was obtained verbally from patients when possible, and opt‐out was applied when consent could not be provided due to the patient's condition or death at the time of registration. Patients were anonymized using a unique study identification code, which remained unchanged throughout the study. The study was approved by the institutional review boards at each of the six participating hospitals.

Patients aged ≥ 18 years with previously untreated CD30‐positive ATL who received BV‐CHP were included in the study. The exclusion criterion was patients deemed ineligible by the investigator; presence of hypercalcemia, and organ dysfunctions such as renal and liver dysfunctions, were not defined as exclusion criteria. The study enrolled all patients who met the eligibility criteria at participating sites and had completed at least 4 weeks since discontinuation or completion of BV‐CHP treatment.

### 2.2 Endpoints

The primary endpoint was the ORR for patients with untreated CD30‐positive ATL receiving BV‐CHP. The ORR was defined as the proportion of patients achieving a complete response (CR), unconfirmed CR (CRu), or partial response (PR) from the start of treatment until discontinuation. Response and progression were assessed using the ATL response criteria from the 2009 international consensus report [4].

Secondary endpoints included OS, measured from treatment initiation to the date of death from any cause; PFS, defined as the time from treatment initiation to disease progression or death; complete response rate (CRR), representing the proportion of patients achieving CR or CRu; response rate by lesion site; disease control rate (DCR), representing the proportion of patients with CR, CRu, PR, and stable disease (SD); time to treatment failure (TTF), time to next treatment (TTNT), the duration until treatment discontinuation, exacerbation, or death; relative dose intensity (RDI); and safety information.

### 2.3 Statistical Analysis

All statistical analyses were conducted using SAS 9.4 or higher, employing a two‐sided test at a significance level of 5%. Categorical data were summarized using frequency and percentage, while continuous data were reported as descriptive statistics.

For the primary endpoint, ORR was calculated as the frequency and percentage of responses in the best overall and final responses, with 95% exact confidence intervals (CIs) determined using the Clopper‐Pearson method. Subgroup analyses were conducted by lesion site (lymph nodes, skin, and peripheral blood), ATL subtype (acute and lymphoma), age group (≤ 70 years and > 70 years), and CD30 details. For secondary endpoints, OS and PFS were assessed using the Kaplan‐Meier method to calculate median values and their 95% CIs. CRR and DCR were also determined using the Clopper‐Pearson method. TTF and TTNT were calculated using the Kaplan‐Meier method, with frequencies of relevant events presented. RDI was calculated for each of the four drugs (brentuximab vedotin, cyclophosphamide, doxorubicin, and prednisolone), with descriptive statistics reported [24]. AEs were summarized by incidence rates, with 95% CIs, and coded to a System Organ Class and Preferred Term using the Medical Dictionary for Regulatory Activities Version 26.1 or higher. Presence or absence of relapse of ATL was also summarized.

## 3 Results

### 3.1 Study Population

**FIGURE 1:**
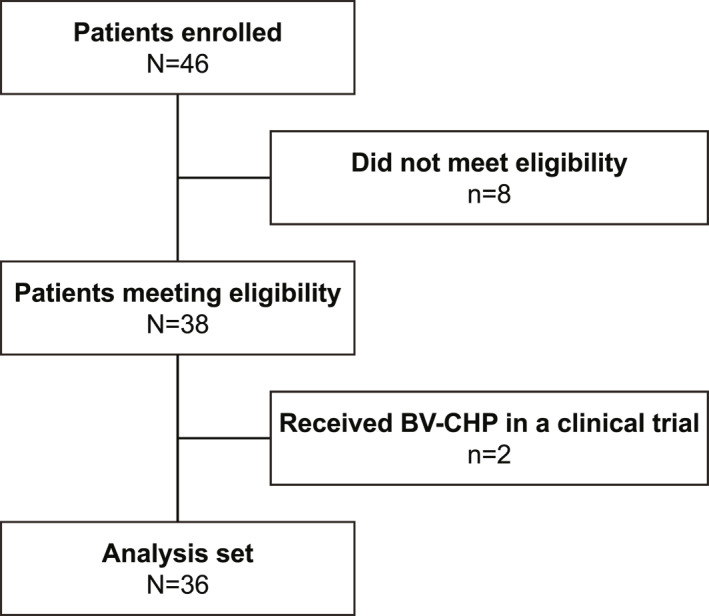
Patient disposition flowchart. BV‐CHP, brentuximab vedotin, cyclophosphamide, doxorubicin, and prednisolone.

Clinical data were collected from six hospitals. Out of 46 patients enrolled, eight were excluded for not meeting the eligibility criteria, and two additional patients were excluded for receiving BV‐CHP treatment in a clinical trial, leaving 36 patients for analysis (Figure 1). These 36 patients represent those who received BV‐CHP treatment from April 2020 to January 2024.

The median age was 71 years (range: 53–92), and 66.7% (24/36) were female (Table 1). For ATL subtype, 52.8% (19/36) of patients were classified as having acute‐type and 47.2% (17/36) as lymphoma‐type. Most patients (32/36) were at an advanced stage of disease, with 25.0% (9/36) at Stage III and 63.9% (23/36) at Stage IV. For the simplified ATL prognostic index [25], 27.8% (10/36) of patients were categorized as low‐risk, 50.0% (18/36) as intermediate‐risk, and 16.7% (6/36) as high‐risk. The median follow‐up time for the patients was 372.5 days (range: 74–1253).

**TABLE 1:** Patient demographics and baseline characteristics.

Characteristic	Analysis set (*N* = 36)
Age, years	
Median (range)	71 (53–92)
≤ 70	13 (36.1)
> 70	23 (63.9)
Sex	
Male	12 (33.3)
Female	24 (66.7)
ECOG performance status	
0	16 (44.4)
1	16 (44.4)
2	4 (11.1)
3	0
4	0
ATL subtype	
Acute	19 (52.8)
Lymphoma	17 (47.2)
Ann Arbor clinical stage	
I	0
II	2 (5.6)
III	9 (25.0)
IV	23 (63.9)
Unknown	2 (5.6)
Simplified ATL‐PI	
Low risk	10 (27.8)
Intermediate risk	18 (50.0)
High risk	6 (16.7)
Unknown	2 (5.6)
CD30 expression^a^, %	
Median (range)	40.8 (7.4–91.4)
< 10%, n	1
≥ 10%, n	19

*Note:* Data are *n* (%) unless otherwise specified.

Abbreviations: ATL, adult T‐cell leukemia‐lymphoma; ATL‐PI, prognostic index for acute and lymphoma ATL; ECOG, Eastern Cooperative Oncology Group; FCM, flow cytometry; IHC, immunohistochemistry.

^a^ In all patients, CD30 positivity was confirmed through either IHC or FCM; the quantitative results from the 20 patients measured using FCM showed a CD30 positivity rate of 40.8%.

CD30 positivity was confirmed in all 36 patients through IHC (*n* = 22) or FCM (*n* = 14). Quantification of CD30 positivity (*n* = 20) yielded a median expression level of 40.8% (range: 7.4–91.4); one patient had CD30 expression < 10%, and 19 patients had CD30 expression ≥ 10%.

### 3.2 Effectiveness

The median number of cycles of BV‐CHP treatment was three (range: 1–6). The median RDI of BV was 86.9% (range: 57.8–123.5).

ORR was 86.1% (31/36) (95% CI 70.5–95.3), consisting of 27.8% (10/36) of patients with CR, 33.3% (12/36) with CRu, and 25.0% (9/36) with PR (Table 2). CRR was 61.1% (22/36) (95% CI 43.5–76.9), while DCR was 91.7% (33/36) (95% CI 77.5–98.3). At baseline, 32 of the 36 patients had lymph node lesions, 11 had ATL cells circulating in peripheral blood, and six had skin infiltration. The ORR for lymph nodes, peripheral blood, and skin was 93.8% (30/32), 90.9% (10/11), and 83.3% (5/6), respectively. The ORR for acute and lymphoma ATL subtypes was 78.9% (15/19) and 94.1% (16/17), respectively. When comparing ORR between patients aged ≤ 70 years versus those > 70 years, rates were similar, with an ORR of 84.6% (11/13; CRR 69.2% [9/13]) in the younger group compared with 87.0% (20/23; CRR 56.5% [13/23]) in the older group. For CD30‐positivity subgroups (*n* = 20), one patient with a CD30 expression < 10% had a CR; of the patients with a CD30 expression ≥ 10%, three had a CR, five had a CRu, and six had a PR, yielding an ORR of 73.7% (14/19).

**TABLE 2:** Summary of response at the end of treatment.

Response^a^	Analysis set (*N* = 36)	Lesion site			ATL subtype	
		Lymph nodes (*N* = 32)	Blood (*N* = 11)	Skin (*N* = 6)	Acute (*N* = 19)	Lymphoma (*N* = 17)
Overall response rate	31 (86.1)	30 (93.8)	10 (90.9)	5 (83.3)	15 (78.9)	16 (94.1)
Response^b^						
CR	10 (27.8)	12 (37.5)	4 (36.4)	5 (83.3)	3 (15.8)	7 (41.2)
CRu	12 (33.3)	13 (40.6)	0	0	5 (26.3)	7 (41.2)
PR	9 (25.0)	5 (15.6)	6 (54.5)	0	7 (36.8)	2 (11.8)
SD	2 (5.6)	1 (3.1)	1 (9.1)	0	2 (10.5)	0
PD	2 (5.6)	0	0	0	1 (5.3)	1 (5.9)
NE	1 (2.8)	1 (3.1)	0	1 (16.7)	1 (5.3)	0

*Note:* Data are *n* (%).

Abbreviations: ATL, adult T‐cell lymphoma‐leukemia; CR, complete response; CRu, unconfirmed complete response; JCOG, Japan Clinical Oncology Group; NE, not evaluable for response; PD, progressive disease; PR, partial response; SD, stable disease.

^a^ Best overall response was determined by the best overall effect through the efficacy evaluation.

^b^ Response and progression were assessed using the JCOG version of the ATL response criteria.

**FIGURE 2:**
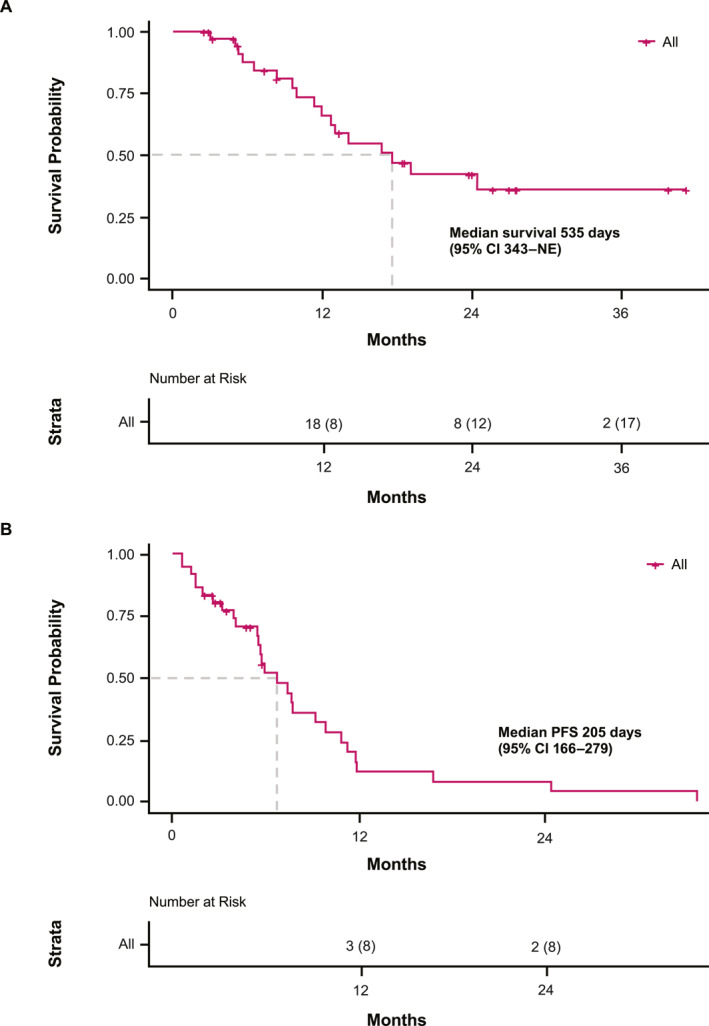
Survival in the analysis set. (A) Overall survival. (B) Progression‐free survival. CI, confidence interval; NE, not estimable; PFS, progression‐free survival.

OS and PFS are shown in Figure 2. The median OS was 535 days (95% CI 343–not estimable). The median PFS was 205 days (95% CI 166–279), with a median TTF of 166 days (95% CI 91–205).

**FIGURE 3:**
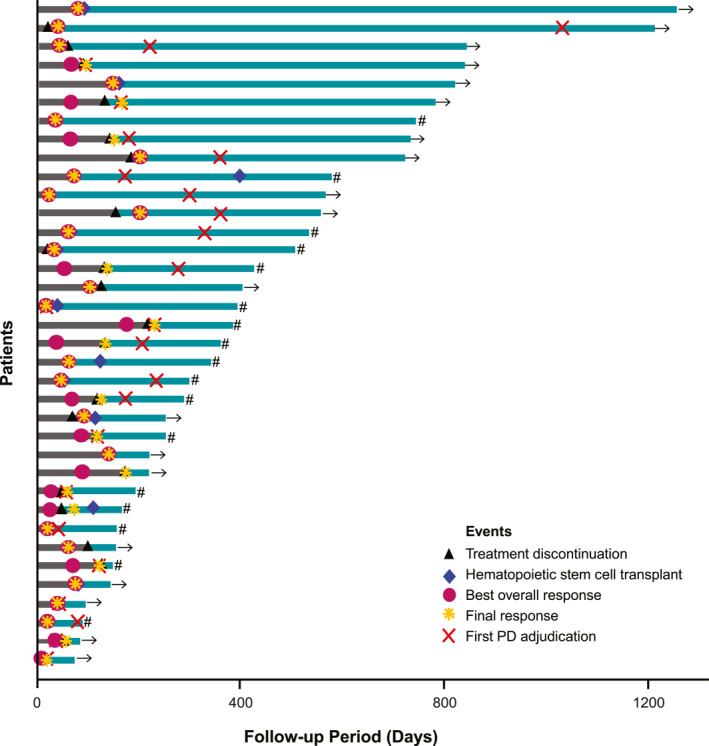
Swimmer plot of clinical course for individual patients. The period highlighted in dark gray on the bar represents the BV‐CHP administration period, and the remaining periods are highlighted in teal. If the patient is censored, it is marked as →, and if the patient has died, it is marked as ‘#’. BV‐CHP, brentuximab vedotin, cyclophosphamide, doxorubicin, and prednisolone; PD, progressive disease.

The median timing for the next treatment was 107 days (95% CI 69–138). The clinical course for each patient is shown in Figure 3. Of the 36 patients in the analysis set, 21 experienced a relapse.

### 3.3 Safety

Treatment‐emergent AEs (TEAEs) of any grade and grade ≥ 3 were both observed in 88.9% (32/36) of patients (Table 3). The most common hematological TEAE of any grade, occurring in ≥ 10% of the patient population, included neutropenia (77.8%; 28/36), febrile neutropenia (38.9%; 14/36), thrombocytopenia (19.4%; 7/36), and anemia (11.1%; 4/36). Among non‐hematological TEAEs of any grade reported in ≥ 5% of patients, peripheral neuropathy was observed in 11.1% (4/36) of patients, herpes zoster in 8.3% (3/36), and COVID‐19 infection, CMV infection, sepsis, and interstitial lung disease in 5.6% (2/36) of patients for all instances. While peripheral neuropathy events were identified in four patients (three with grade 2 and one with grade 1), none progressed to grade 3 or higher.

**TABLE 3:** Summary of common AEs.

Details	Analysis set (*N* = 36)	
	Any grade^a^	Grade ≥ 3
Patients with any TEAEs	32 (88.9)	32 (88.9)
Hematological		
Neutropenia^b^	28 (77.8)	28 (77.8)
Febrile neutropenia	14 (38.9)	14 (38.9)
Thrombocytopenia^c^	7 (19.4)	4 (11.1)
Anemia	4 (11.1)	1 (2.8)
Leukopenia	2 (5.6)	1 (2.8)
Non‐hematological		
Peripheral neuropathy^d^	4 (11.1)	0
Herpes zoster	3 (8.3)	2 (5.6)
COVID‐19 infection	2 (5.6)	2 (5.6)
CMV infection^e^	2 (5.6)	2 (5.6)
Sepsis	2 (5.6)	2 (5.6)
Interstitial lung disease	2 (5.6)	1 (2.8)

*Note:* AEs were coded to a System Organ Class and Preferred Term using Medical Dictionary for Regulatory Activities Version 26.1. Data are *n* (%).

Abbreviations: AE, adverse event; CMV, cytomegalovirus; TEAE, treatment‐emergent adverse event.

^a^ Only AEs occurring in ≥ 5% of patients are reported for any grade.

^b^ Includes the Preferred Terms neutropenia and neutrophil count decreased.

^c^ Includes the Preferred Terms thrombocytopenia and platelet count decreased.

^d^ Includes the Preferred Terms peripheral neuropathy and peripheral sensory neuropathy.

^e^ Includes the Preferred Terms cytomegalovirus gastrointestinal infection and pneumonia cytomegaloviral.

Of the 36 patients, seven discontinued BV treatment, and one had their dosage of BV adjusted due to AEs. Of the patients with peripheral neuropathy events, one had their BV dosage adjusted because of AEs. All AEs resolved or improved over time, and there were no treatment‐related deaths.

### 3.4 Impact of BV‐CHP Before Allogeneic Stem Cell Transplantation

Among the 36 patients, 11 underwent allogeneic stem cell transplantation (10 received umbilical cord blood transplants, and one received a peripheral blood stem cell transplant). The median age of these patients was 65 years (range: 61–72), compared with a median age of 75 years (range: 53–92) for those who did not undergo transplantation. The disease status at the time of transplantation included 18.2% (2/11) of patients who underwent transplantation with CR, 72.7% (8/11) with PR, and 9.1% (1/11) with SD, following a median of three cycles of BV‐CHP (range: 1–6).

The median PFS following the initiation of BV‐CHP was 234 days (95% CI 168–343) for patients who underwent allogeneic stem cell transplantation, while patients without transplantation had a median PFS of 180 days (95% CI 96–279).

Acute graft‐versus‐host disease (GVHD) was observed in two of the 11 patients who underwent allogeneic stem cell transplantation, with one case classified as grade 1 and the other as grade 4. Chronic GVHD was not observed in these 11 patients.

## 4 Discussion

This study represents the first multicenter real‐world analysis to evaluate BV‐CHP as a first‐line treatment for CD30‐positive ATL. The primary objective was to assess the effectiveness of BV‐CHP in CD30‐positive previously untreated patients with ATL in Japan. A key finding of this study is that BV‐CHP demonstrated high effectiveness, with an ORR of 86.1%, and a manageable safety profile, with no new safety signals observed.

Mogamulizumab has demonstrated efficacy in combination with the mLSG15 regimen. In a Phase 2 trial of patients with CCR‐4‐positive aggressive ATL, mogamulizumab plus mLSG15 achieved an ORR of 86% and a median PFS of 8.5 months, an improvement from an ORR of 75% and a median PFS of 6.3 months reported with mLSG15 alone [21]. These favorable results have led to this combination being recommended as a treatment in the Japanese Society of Hematology guidelines [26]. Yoshimitsu et al. reported that the combination of mogamulizumab with CHOP demonstrated significantly improved PFS in elderly patients who were ineligible for transplantation [22]. The 1‐year PFS was 36.2%, and CR and ORR were 64.6% and 91.7%, respectively. In comparison, BV‐CHP in our study demonstrated an ORR of 86.1% and a median PFS of 6.7 months (205 days) in a slightly younger patient group than those treated with mogamulizumab plus CHOP (median age: 71 vs. 74 years), indicating comparable effectiveness of BV‐CHP to mogamulizumab plus mLSG15. Additionally, BV‐CHP demonstrated higher ORR values than biweekly CHOP (66%) and azidothymidine (AZT)‐interferon alfa (IFN) regimens (71%) [18, 27], with the latter often used in the USA and Europe for acute‐type ATL but not for lymphoma‐type ATL [28].

Our study showed comparable results to recent findings from Dittus et al., who reported an ORR of 87.5% and a median PFS of 7.11 months in their analysis of patients with aggressive ATL treated with > 2 cycles of brentuximab vedotin combined with cyclophosphamide, doxorubicin, etoposide, and prednisone (BV‐CHEP) [29]. Although differences in patient background and disease subtypes across the clinical trials and real‐world studies present certain limitations, the median PFS of 6.7 months observed in our study further supports BV‐CHP as a viable treatment option for ATL.

In terms of safety, the most frequent grade 3 or higher TEAEs in this study were hematological, including neutropenia, febrile neutropenia, and thrombocytopenia. These were similar to those observed with mogamulizumab plus mLSG15,^21^ and no patients died due to TEAEs in the present study. This demonstrates that the TEAEs with BV‐CHP are manageable. Importantly, even in elderly patients aged ≥ 70 years, the ORR in the present study remained high at 87.0%, with a CRR of 56.5%, suggesting that BV‐CHP is effective across age groups.

Regarding CD30 expression, previous studies have investigated its potential correlation with treatment response in patients with CD30‐expressing lymphomas, such as Hodgkin lymphoma, PTCL, and cutaneous T‐cell lymphoma (CTCL). One study reported that patients with CTCL and CD30 expression levels < 5% had a lower likelihood of responding to treatment compared with those with ≥ 5% CD30 expression [30]. However, the majority of other studies in both T‐cell and B‐cell non‐Hodgkin lymphoma, including the pivotal ECHELON‐2 trial [23], have shown no significant correlation between CD30 expression levels and treatment efficacy [31, 32, 33]. In the context of ATL, there have been no comprehensive reports analyzing the correlation between CD30 expression and the effectiveness of BV. While the sample size in our study is relatively small, we observed no clear relationship between the level of CD30 expression and treatment outcomes in patients with ATL treated with BV‐CHP. This suggests that BV‐CHP may be effective in patients with ATL regardless of CD30 expression levels, although further studies with larger patient cohorts are required to validate this observation.

Another important aspect of this study was the impact of BV‐CHP on allogeneic stem cell transplantation, which represents the only potentially curative treatment for aggressive ATL [34, 35]. It has been reported that chemotherapy combined with mogamulizumab increases the incidence of GVHD prior to transplantation [36], and such combination therapy will inevitably be considered in cases where transplantation is planned. In our cohort, 11 out of 36 patients underwent transplantation after BV‐CHP treatment. Two patients developed GVHD, with one case being grade 1 and the other grade 4, both of which were manageable. There were no treatment‐related deaths within 100 days post‐transplant, and the only early death was due to ATL progression (data not reported). To date, the impact of BV followed by transplantation has not been elucidated in ATL, but BV prior to transplantation in patients with Hodgkin lymphoma was not associated with and increase the incidence of acute GVHD and was linked to a lower risk of chronic GVHD [37]. Moreover, although the number of cases is small, our findings indicate that BV‐CHP does not negatively impact the outcomes of allogeneic stem cell transplantation and can be used as a bridge to transplantation.

A key strength of this study is that it included elderly patients aged ≥ 70 years, which provides insights into the effectiveness and safety of BV‐CHP in a typically underrepresented age group. By including elderly patients, this study provides new data on the effectiveness and safety of BV‐CHP compared with other reports. Additionally, it is the first study to examine the effectiveness and safety of BV‐CHP in a larger sample of previously untreated patients with ATL.

This study has several limitations due to its retrospective design. Firstly, missing or incomplete data, which could not be supplemented, may have been present because data collection relied solely on past medical records and may have potentially affected the accuracy of the results. Secondly, data were contributed only by participating physicians, which may not fully represent all sites, introducing potential selection bias. Variations in the quality of physician documentation could also impact data consistency. Despite efforts to reduce selection bias by including all eligible patients who had completed BV treatment for over 4 weeks, these limitations should be considered when interpreting the findings. Importantly, because PFS and OS were not censored for patients who underwent allogeneic stem cell transplantation, PFS and OS may be overestimated, and therefore both should be interpreted with caution.

## 5 Conclusion

This multicenter retrospective study showed that BV‐CHP is an effective first‐line treatment for CD30‐positive ATL, with a high ORR and manageable safety profile. No new safety signals were identified, and the treatment appeared to be effective across different age groups and ATL subtypes. The results of this study suggest that BV‐CHP could become a potential standard of care for ATL.

## Author Contributions

All authors participated in the interpretation of study results, and in the drafting, critical revision, and approval of the final version of the manuscript. Takahiro Yoshida, Kiyoshi Okazuka, and Atae Utsunomiya were involved in the study design and statistical analyses. Masahito Tokunaga, Junya Makiyama, Motoaki Shiratsuchi, Takanori Toyama, Satoshi Oka, and Ilseung Choi were investigators in the study and participated in data collection.

## Ethics Statement

The study was conducted in accordance with the Ethical Guidelines for Medical and Biological Research Involving Human Subjects. The study was approved by the institutional review boards at each of the six participating hospitals.

## Consent

Informed consent was obtained verbally from patients when possible, and opt‐out was applied when consent could not be provided due to the patient's condition or death at the time of registration. Patients were anonymized using a unique study identification code, which remained unchanged throughout the study.

## Conflicts of Interest

Masahito Tokunaga has received honoraria from Takeda Pharmaceutical Company Limited. Junya Makiyama has served as a consultant for Janssen Pharmaceutical and Takeda Pharmaceutical Company Limited, received honoraria for lectures, presentations, or speakers bureaus from AbbVie GK, AstraZeneca, Bristol‐Myers Squibb, Chugai Pharmaceutical, Daiichi Sankyo Company, Janssen Pharmaceutical, Kyowa Kirin, Meiji Seika Pharma, Ono Pharmaceutical, Otsuka Pharmaceutical, Sanofi, SymBio Pharmaceuticals, and Takeda Pharmaceutical Company Limited, and received support for attending meetings and/or travel from Takeda Pharmaceutical Company Limited. Satoshi Oka has received honoraria from AbbVie GK, Bristol‐Myers Squibb, Janssen Pharmaceutical, Meiji Seika Pharma, Nippon Shinyaku, Pfizer, and Takeda Pharmaceutical Company Limited. Takahiro Yoshida and Kiyoshi Okazuka are employees of Takeda Pharmaceutical Company Limited. Atae Utsunomiya has served as a consultant for HUYA Japan, JIMRO, and Meiji Seika Pharma, and has received honoraria from Bristol‐Myers Squibb, Daiichi Sankyo Company, Kyowa Kirin, Meiji Seika Pharma, and Takeda Pharmaceutical Company Limited. Motoaki Shiratsuchi, Takanori Toyama, and Ilseung Choi have no conflicts of interest to disclose.

## Peer Review

The peer review history for this article is available at https://www.webofscience.com/api/gateway/wos/peer-review/10.1002/hon.70141.

## Data Availability

The datasets generated during and/or analyzed during the current study are available from the corresponding author on reasonable request.
